# New Appliances and Things Medical

**Published:** 1907-03-16

**Authors:** 


### NEW APPLIANCES AND THINGS MEDICAL.

[We shall be glad to receive, at our Office, 28 & 29 Southampton Street, Strand, London, W.C., from the manufacturers, specimens of all new preparations and appliances which may be brought out from time to time.]

### A NEW HOSPITAL DRESSING TABLE.

(MESSRS. WITT, CUXSON AND CO., 292 BURBURY STREET, BIRMINGHAM.)

WE have had the opportunity of inspecting a model of a hospital dressing table designed and made by this firm, and which is used with great satisfaction in the London Hospital. It is constructed of seamless steel tubing, riveted and brazed together in such a fashion as to make it perfectly rigid, combining in this way strength with lightness. This table has an adjustable and detachable douche carrier which enables it to be used not only as a ward table for dressings, but for irrigating purposes. This is a new and highly commendable method. In addition to this the table is fitted with removable Witcux metal shelves, which have the inestimable advantage of not permitting medicine-bottles, or bottles containing lotions from slipping down and breaking, as is the case where glass shelves are used. The metal shelves have a further advantage over glass of being durable and not liable to break and split and crack at the edges. The table is non-corrosive and resists all acid solutions. They are made by this firm in various sizes, suitable for different wards, and are fitted with ball-bearing castors, which enables the table to be moved noiselessly about the wards with the greatest ease. The metal of which this table is made is specially recommended for the tops of ward and other lockers, for shelves in operating theatres, and when tinned for sterilising boxes.

